# Realizing and Allocating Savings From Improving Health Care Quality and Efficiency

**Published:** 2010-08-15

**Authors:** Daniel M Fox

**Affiliations:** Milbank Memorial Fund

## Abstract

International efforts to increase the quality and efficiency of health care services may be creating financial savings that can be used to improve population health. This article examines evidence that such savings (ie, a quality/efficiency or value dividend) are accruing and how they have been allocated and assesses the prospects for reallocating future savings to improve population health. Savings have resulted mainly from reducing the number of inappropriate or harmful interventions, managing care of people with chronic disease more effectively, and implementing health information technology. Savings to date have accrued to the revenues of public and private collective purchasers of care and large provider organizations, but none seem to have been reallocated to address other determinants of health. Furthermore, improved quality sometimes increases spending.

## Introduction

The rapid growth of an international movement to improve the quality (including the safety) and efficiency of health care services has led to speculation about whether any resulting savings can be used to improve population health. This article explores the limited evidence about whether improvements in the quality and efficiency of health care services yield net savings (ie, a quality/efficiency or value dividend) and scantier evidence about how savings to date have been allocated.

The possibility that a portion of any dividend from improving the quality and efficiency of health care services can be used to address other determinants of health has recently attracted interest in several industrial countries that provide universal coverage. A select committee of the British Parliament recommended in 2007 that the National Institute of Health and Clinical Excellence (NICE) offer more guidance about what health services to "disinvest" from and how to reinvest the savings in clinical and community health interventions. Australian researchers recently proposed criteria for disinvestment and reinvestment by government health agencies and documented support for such a program among policy makers (1). Donald Berwick, an American who is an international leader in quality improvement, argues on the basis of international experience that it is feasible to achieve the "triple aim" of "improving the experience of care, improving the health of populations, and reducing per capita costs of health care" (2).

## Searching for a Quality/Efficiency Dividend in the United States

The search for a dividend as a result of improving the quality (including safety) and efficiency of health care services in the United States began in the 1980s. Expenditures for health care had been increasing for several decades at a rate higher than general inflation. By the end of the 1970s, most policy makers for health care had concluded that any expansion of access would require slowing the rate of increase in spending.

The recession of the early 1980s exacerbated concern among employers and union leaders that the international competitiveness of American industry was declining for reasons that included employment-based health care coverage. To address this decline in competitiveness, American corporations reimported from Japan techniques of scientific management that had originated in the United States earlier in the century. Business leaders applied these techniques to all aspects of their business, including spending for health services.

Executives and physician leaders of large health provider systems also accorded considerable attention to what would soon be called *quality improvement science*. Managers of hospitals and health systems had begun in the 1970s to identify with private sector executives rather than with their predecessors, for whom careers in health care were extensions of philanthropic service or public administration. These managers were particularly aware of the increasing number of their patients who were covered by the self-insured benefit plans of large firms that engaged in formal quality improvement. As a result of incentives in the Employee Retirement Income Security Act of 1974 (ERISA), more than half of workers and their dependents were enrolled in these plans by the late 1980s.

Policy makers for health care in the federal government joined the quality improvement movement during the Reagan administration, when the Health Care Financing Administration (HCFA, now the Centers for Medicare and Medicaid Services) imposed prospective payment for Medicare Part A (hospital) benefits. Disease related groups (DRGs), the regulatory tool for prospective payment, had been devised to improve efficiency and quality by measuring how hospitals used resources. The co-investigator for the research project that conceptualized DRGs, John D. Thompson, was strongly influenced by analytical methods to improve quality and efficiency in hospitals that Florence Nightingale had devised in the 1850s and 1860s (3). As a result, DRGs had a dual purpose from their introduction into policy: to contain the growth of public spending and to create incentives to reduce the average length of hospital stays and the overuse of ancillary services.

HCFA addressed quality more explicitly during the second Reagan administration. In 1986 it began a controversial project that compared, and published, death rates among hospitals. A year later the administrator of HCFA, William Roper, was lead author of an article in the *New England Journal of Medicine* that advocated measuring the effectiveness of health services to pay, eventually, for what worked. In response to the Omnibus Budget Reconciliation Act of 1987, HCFA and external researchers, led by John Morris, devised what became the minimum data set for measuring and reporting the quality of care in residential nursing facilities.

Beginning in the late 1980s, states used their authority to regulate health plans and facilities to encourage transparency about outcomes and quality. Public agencies in New York and Pennsylvania, for example, compared death rates of hospital patients who had cardiac surgery. In California, a new public agency collected information from hospitals, including data about outcomes. Many states required health plans to make public the data they had reported to the National Committee on Quality Assurance, a nonprofit organization.

The measurement of quality in clinical practice and the dissemination of techniques to improve it accelerated during the 1990s. The Institute for Healthcare Improvement trained, advised, and inspired many health care professionals and leaders of provider systems. Managed care plans used evidence about quality to select clinicians and hospitals for their networks. They used the controversial methods of managed care to control costs by increasing efficiency as well as by curtailing use.

In parallel with the quality improvement movement, researchers were collaborating internationally to improve methods for evaluating the effectiveness and efficiency of health care technology and care processes. Systematic reviews were a powerful tool for identifying bias in research about interventions and then pooling data from multiple studies to increase statistical power. Eighty-seven systematic reviews appeared in the international literature in 1988, the year before publication of the first set of reviews evaluating an entire field of care. During the next 2 decades the number of new and updated systematic reviews published each year grew to more than 2,500. Moreover, by the first decade of the 21st century an increasing number of reviews were comparing the effectiveness of competing interventions. During the same years, advances in methods of improving health services occurred in the disciplines of economics and decision science. Perhaps most important, the evolving methods of analyzing cost-effectiveness yielded more precise estimates of relative value for money.

Insurance plans and public agencies increasingly used findings from research on effectiveness and efficiency to inform decisions about coverage. The Blue Cross/Blue Shield Association created a program to assess health technology in 1985, building on work it began in the 1970s. Other organizations, commercial and nonprofit, provided technology assessment to provider organizations by subscription. A new international organization, the Cochrane Collaboration, set standards for, produced, and published systematic reviews. The federal Agency for Healthcare Research and Quality (under an earlier name) began in 1997 to commission research evaluating the effectiveness of interventions from organizations it designated evidence-based practice centers (4).

A committee of the Institute of Medicine shocked the health sector and the media in 2000 when it estimated that 80,000 to 100,000 unnecessary deaths occurred in hospitals each year. A year later, the committee published recommendations for "crossing the quality chasm," revealed by these deaths and other evidence of inadequate care (5).

By the turn of the new century, the rapidly evolving methods for measuring and improving quality and evaluating the effectiveness and comparative effectiveness of interventions were informing policy and practice in the United States and other industrial countries. The chief medical officers of integrated delivery systems and many other large provider organizations urged greater use of what was commonly (if controversially) called evidence-based health research in clinical decisions. The Veterans Health Administration had, since 1993, begun to make significant and widely publicized improvement in quality under the leadership of Ken Kizer. Berwick and the Institute for Healthcare Improvement stimulated and documented quality improvement as a result of "learning collaboratives" of physicians and hospital staff (6).

In 2001, states began to use evidence of comparative effectiveness to establish formularies, called preferred drug lists (PDLs), for Medicaid and other public programs. Three states began collaborating in 2003 to commission, finance, and make publicly accessible systematic reviews of drugs in particular classes. The number of collaborators had grown to 17 by 2009 and included a Canadian intergovernmental agency. Forty-five states had PDLs in 2009. Evidence accumulated that research-based PDLs improved quality and controlled the growth of cost (7).

But much evidence of the effect of other quality improvement activities on expenditures was inconclusive. In the 1990s, most practitioners of quality improvement and evidence-based health research prioritized improving outcomes over cost savings. Nevertheless, in 1998 Shortell and colleagues cited several reports of savings as a result of continuous quality improvement. Intermountain Health Care, for example, reported $30 million of annual savings from "60 ongoing clinical improvement initiatives." Most of the studies the authors located, however, assessed evidence from a single site and used "relatively weak" designs, primarily "before-and-after observations" (8).

Five years later, in an article that has been cited frequently, Sheila Leatherman and colleagues asked whether "improving health care quality cost money or save[d] money." The authors concluded that "even where analyses do exist, the answer varies with the stakeholder's viewpoint and the time frame examined" (9).

Subsequent research, especially in the United Kingdom, documented that improving quality sometimes led to improved outcomes and fewer adverse events but at additional cost (10). The chairman of NICE emphasized in 2009, for example, that "in practice [NICE guidelines] tend to add to the cost of providing care" (11).

## The Current Search for Savings

Little evidence shows that improving quality and efficiency in clinical settings yields savings that are large and sufficiently identifiable to be reallocated. In 2003 Leatherman and colleagues described 3 perspectives for linking quality and cost: business, economic, and social (9). Under their definition, a business case for savings would be made if providers realize a return on their investment in a reorganized care process in a "reasonable time frame." An economic case would be persuasive if "discounted financial benefits exceed discounted costs, whether they accrue to patients, employers, providers or payers." A social case would be evidence of any "benefit to the individual (patient) or to society of improved health status and productivity, regardless of cost."

Leatherman and other colleagues subsequently documented the weakness of the business case for quality and efficiency. In 2005 they reviewed and summarized articles in the American literature that contained sufficient data to calculate a return on investment to providers. They found only 15 articles that met their inclusion criteria and concluded that "scant attention is currently paid in the quality-of-care literature to the cost of implementing quality-enhancing interventions" (12).

In 2008 Leatherman, again with other colleagues, reported on a "demonstration project designed to measure the business case for selected quality interventions in high-risk high-cost populations in Medicaid managed care organizations." They concluded that savings would result mainly from interventions "that have potential for short-term return on investment and primarily seek to reduce avoidable emergency room and inpatient hospital utilization." They warned, however, that managed care organizations would be wary of quality improvement that achieved savings because Medicaid agencies might reduce capitation rates as costs declined (13).

In contrast, the Center for Health Care Strategies (CHCS) argues that the interests of Medicaid agencies and managed care organizations can be aligned. CHCS has devised and, in collaboration with the Commonwealth Fund, is promoting tools with which state Medicaid programs can conduct "return on investment analysis" to "lower costs without sacrificing quality of care or enrollment capacity" (14).

Elliott Fisher and colleagues recommend policy to achieve savings linked to quality improvement on the basis of their research at Dartmouth on unwarranted regional variation in the use of health care. Their studies have documented "marked regional differences in spending [for Medicare] . . . after careful adjustment for health." Because integrated delivery systems "offer great promise for improving quality and lowering costs," Medicare policy should foster "local organizations' accountability for quality and costs through performance measurement and shared savings payment reform." The savings would be shared among physicians and health systems. This proposal has attracted considerable attention in the media and among policy makers because Fisher and colleagues estimate that approximately 30% of Medicare spending is unnecessary (15).

Researchers at the RAND Corporation reached a similar conclusion, using different methods. A RAND report of 2005, still quoted by the media in 2009, estimated that substantial savings would result from improved quality and efficiency. RAND researchers estimated that if 90% of hospitals and physicians adopted health information technology, the combined savings from improved health, safety, and efficiency would during the next 15 years total approximately 6% of 2009 spending for health care.

Other researchers are less optimistic about potential savings from avoiding the overuse, misuse, or inappropriate use of care. Bentley and colleagues, for example, devised a "typology of operational waste," which they define as duplication of services, inefficient processes, overly expensive inputs, and "quality defects that result in rework or scrapping." They found that such waste amounted only to 1.9% to 3.4% of US health care spending in 2006. They also found it difficult to "identify clinical procedures that are *unambiguously* wasteful" (16).

Other recent studies found only limited savings as a result of improving the coordination of care (17). A 2007 study of countries that are members of the Organisation for Economic Co-operation and Development described evidence of "cost efficiency" as a result of better coordination as "inconclusive" (18). A review of 15 randomized trials of the effects of care coordination on hospitalization, quality of care, and health expenditures among Medicare beneficiaries concluded that, "Coordination programs without a strong transitional care component are unlikely to yield net Medicare savings" (19).

Some experts emphasize political and cultural barriers to accruing savings by reducing the volume of ineffective care. Bryan and Graeme Haynes, for example, listed many interventions (eg, use of antioxidants for the prevention of cancer and cardiovascular disease) that are still used although persuasive research has demonstrated that they offer no benefits or can be harmful. Then they describe how "vested interests" work to "make us forget that the justification for their promotion has been gored" (20).

Anecdotal evidence, however, continues to encourage optimism about generating a value dividend, despite the discouraging research findings I have surveyed. Large provider organizations, for example, report savings as a result of quality improvement in particular service lines. Examples include Ascension Health, the Geisinger Health System, Sutter Health, and Kaiser Permanente. Many experts on quality improvement claim that the Swedish county of Jönköping is achieving the lowest per capita costs and highest quality among jurisdictions in that country.

## Conclusion

Both research and anecdotes support the generalization that any dividend that has accrued to date has reduced costs mainly for public purchasers, health plans, and provider organizations. Moreover, such savings have improved the general revenue of these organizations instead of having been reallocated for particular purposes.

There is persuasive evidence, for instance, that many American states are achieving substantial savings in spending for pharmaceutical drugs in public programs by using PDLs that rely on systematic reviews. These savings offset other expenditures for Medicaid and the health benefits of public employees (7). The state of North Carolina is an exception. Under its Community Care program, in statewide operation since 2005, case managers and physicians collaborate to "improve and coordinate care across 1,200 medical practices serving more than 884,000 Medicaid recipients." The state allocates savings achieved by the program to hiring additional staff for the 14 regional networks that administer it (21).

Even in countries with universal coverage and strong commitment to addressing broad determinants of population health, savings from improving value accrue mainly to general revenue. A senior official in Jönköping, replying to my question about the allocation of savings that he estimated to be 2% of the county's health expenditures, wrote: "Our savings go directly to pensions, investments and improvement work, so they are hard to put the finger on as 1 single thing" (personal communication, 2009). Similarly, there is no evidence that savings in Britain, as a result of the implementation of findings from studies conducted by NICE, have been allocated for purposes other than health care.

A recent study explored the feasibility of reallocating resources from health care in Amsterdam to "sustained population-wide health improvement." The authors found that the "municipality held a public health perspective but did not use it to really govern the health system." The sickness fund with the largest market share "had no interest in targeting healthcare to the needs of the Amsterdam population." An executive of the fund said that "[w]e do not represent public interests! We represent our customers." After reviewing relevant literature in the context of their findings, the authors concluded that, "Population health considerations are not central to European health reforms" (22).

Two economists claim that research in their discipline that purports to inform policy makers about how to create value dividends has, perversely, caused spending to increase. The standard method for economic evaluation of health services, Birch and Gafni argue, leads to "an increase in health care expenditures" rather than to savings as a result of flaws in the standard method for calculating "incremental cost-effectiveness ratios" (ICERs). They propose that, instead of calculating ICERS, purchasing organizations pay for new technologies only when their "adoption leads to an unambiguous increase in health gains from available resources." However, the method they recommend for estimating health gains assumes that policy makers would ration care (by ceilings on resources) and would disinvest from technologies that do not improve health (23).

Other experts doubt that improving overall population health would have the highest priority when a value dividend is reallocated. "Societal goals," Bentley and colleagues write, "override basic cost-effectiveness analysis considerations of cost and value." For example, "as a society we may prefer to provide care to the sickest, most vulnerable patients, even though our money could buy greater improvements in life span or quality of life if used for another purpose" (16). Policy makers are likely, that is, to ration spending to improve overall population health to avoid rationing health care.

Many people steeped in American health politics would likely agree. Any future savings from improving the quality and efficiency of health care in the United States would most likely be allocated to expanding access (best case) or to slowing the inexorable growth of spending (probable case). Like the illusory Cold War or peace dividend that was reinvested in hot wars and homeland security, any dividend from health care could also finance responses to unanticipated epidemics and disasters.

## References

[1] Elshaug AG, Moss JR, Littlejohns P, Karnon P, Merlin TL, Hiller J (2009). Identifying existing health care services that do not provide value for money. Med J Austr.

[2] Berwick DM Nolan TW Whittington J 27 3 2008 759 769 The triple aim: care, health, and cost Health Aff (Millwood) Berwick DM, Nolan TW, Whittington J. The triple aim: care, health, and cost. Health Aff (Millwood) 2008;27(3):759-69. 18474969 10.1377/hlthaff.27.3.759

[3] Thompson JD (1978). Epidemiology and health services administration: future relationships in practice and education. Milbank Mem Fund Q Health Soc.

[4] Blumenthal D, Kilo CM (1998). A report card on continuous quality improvement. Milbank Q.

[5] Institute of Medicine (2001). Crossing the quality chasm: a new health system for the 21st century.

[6] Baker GR, MacIntosh-Murray A, Porcellato C, Dionne L, Stelmacovich K, Born K (2008). High performing health care systems: delivering quality by design.

[7] Fox DM (2010). The convergence of science and governance: research, health policy and American states.

[8] Shortell SM, Bennett CL, Byck GR (1998). Assessing the impact of continuous quality improvement on clinical practice: what it will take to accelerate progress. Milbank Q.

[9] Leatherman S, Berwick D, Iles D, Lewin LS, Davidoff F, Nolan T (2003). The business case for quality: case studies and an analysis. Health Aff (Millwood).

[10] Klein R (2005). A middle way for rationing healthcare resources. BMJ.

[11] Timmins N (2009). The NICE way of influencing health spending: a conversation with Sir Michael Rawlins. Health Aff (Millwood).

[12] Kilpatrick KE, Lohr KN, Leatherman S, Pink G, Buckel JM, Legarde C (2005). The insufficiency of evidence to establish the business case for quality. Int J Qual Health Care.

[13] Greene SB, Reiter KL, Kilpatrick KE, Leatherman S, Somers SA, Hamblin A (2008). Searching for a business case for quality in Medicaid managed care. Health Care Manage Rev.

[14] Hamblin A, Shearer C (2009). Maximizing quality and value in Medicaid: using return on investment forecasting to support effective policymaking.

[15] Fisher ES, McClellan MB, Bertko J, Lieberman S, Lee JJ, Skinner JS (2009). Fostering accountable health care: moving forward in Medicare. Health Aff (Millwood).

[16] Bentley TG, Efros RM, Palar K, Keeler EB (2008). Waste in the US health care system: a conceptual framework. Milbank Q.

[17] Øvretveit J (2009). Does improving quality save money? A review of evidence of which improvements to quality reduce costs to health service providers.

[18] Hoftmacher MM, Hoxley A, Rusticella E (2007). Improved health system performance through better care performance. Working paper no. 30, Directorate for Employment, Labour and Social Affairs.

[19] Peikes D, Chen A, Schore J, Brown R (2009). Effects of care coordination on hospitalization, quality of care, and health care expenditures among Medicare beneficiaries: 15 randomized trials. JAMA.

[20] Haynes B, Haynes GA (2009). What does it take to put an ugly fact through the heart of a beautiful hypothesis?. Ann Intern Med.

[21] Buntin J (2009). Health care comes home. Governing.

[22] Plochg T, Delnoij DM, Hogervorst WV, van Dijk P, Belleman S, Klazinga NS (2006). Local health systems in the 21st century: who cares? An exploratory study on health system governance in Amsterdam. Eur J Public Health.

[23] Birch S, Gafni A (2006). The biggest bang for the buck or bigger bucks for the bang: the fallacy of the cost-effectiveness threshold. J Health Serv Res Policy.

